# The Work of James Edmund Garretson, A.M., M.D., D.D.S.

**Published:** 1897-03

**Authors:** H. H. Burchard


### THE WORK OF JAMES EDMUND GARRETSON, A.M., M.D., D.D.S.[1]

BY H. H. BURCHARD, M.D., D.D.S.

MODERN dentistry has lost another of its architects, oral surgery its founder, and stomatology one of its greatest forces in the death of Professor Garretson.

Entering the ranks of the dental fraternity when it was hovering on the border-land of the learned professions, he saw and seized the elements of greatness residing in it, and from then to the time of his death labored incessantly to endow dentistry with the dignity which he foresaw was to be its ultimate portion.

Beginning in 1859, he brought to the practice of surgery the training and knowledge of the well-equipped dentist, and how well he blended these two aspects of practice, and what he made from them, is seen in a stupendous work on regional surgery which has remained without a rival for twenty-five years.

It is in the last edition of this great work that the present culmination of stomatology is found; and the former editions exhibit the gradual evolution of a professional condition which began in toleration and has grown to a just recognition. Examining the works upon general surgery prior to the appearance of the first edition of the "Oral Surgery," it is seen the surgical armamentarium for use in the special field embraced by Dr. Garretson was of a type strongly suggestive of the Pompeiian relics in the museum of Naples; viewing it as he left it, we find an instrument cabinet replete with devices designed to expedite and render more humane many of the major operations of surgery, and a class of mechanism fitted for use in refined operations, the authorship of which is traceable to the learned gentleman whose death two professions mourn.

The secret of much of Dr. Garretson's success was not in genius, although he was endowed with genius, but resided in work; in an indomitable energy, a keen perception, and a rigid yet luminous reasoning which was brought to bear upon the problems confronting him. Beginning, as this systematic work did, with years of patient and assiduous labor in the anatomical laboratory, no work begun was ever relinquished until the master held within

---

[1] Read before the Academy of Stomatology, January 26, 1897.

his grasp a clear, full, and philosophical comprehension of every factor involved. In any work engaging his attention he was the incarnation of energy, patience, and modesty.

These were the forces brought to bear upon a field of surgery ignored or half taught by the general surgeon and beyond the province or ken of the dental fraternity as it existed before this vivifying element touched it with the wand of possibility, and at times stung it with the lash of criticism.

Before his day stomatology was in an inchoate state. There was a field common to the general surgeon and medical practitioner and to the dentist; touched by all, embraced by none; treated by all, frequently cured by none, he had lived to see the fruition of much of his toil in the development of a new field of special surgery. His innate modesty never permitted him to assume his full measure of credit due him for the part he played in this evolution.

Reviewing his published works from 1863 to 1895, there is a history beginning in a perfect familiarity of the details of anatomy, normal and morbid, and of normal and perverted physiology, which, while keeping pace year by year with the great advances in each of these fields, year by year has been generalized into guiding principles; for the teaching of principles was the key-note to his professional life.

To him no such thing as an unrelated fact existed, each in his mind stood as representative of cause or effect, and through his long and honorable career in medicine every fact, datum, or phenomenon which his experience evolved, or which came under his notice, was carefully placed in its relations with others so that a continuous chain was formed. In his formulations there were and are matters which are seemingly not in perfect harmony with the details of the several sciences involved in medicine, yet it is readily seen that each is eminently fitted to subserve the purpose for which it was designed, to render clear to the minds of students problems which without such aid would be obscure or lead to mental confusion.

His method of teaching was purely inductive, and any means which could lead to the development of the inductive faculty in the students was applied, leaving to them the task and the acquired ability to deduce from general rules the details involved in them.

Under his touch the dry minutiæ of descriptive anatomy were clothed with alluring forms; the mind of the philosopher and poet endowing mere description with forms which chained and charmed the attention of the student. Facts and figures of biology had woven about them such a skill of interpretation that even the laity saw beauty where before was felt aversion.

Pathology and morbid anatomy were painted in such colors that from repugnance grew an absorbing attention which surrounded even a malignant growth with an interest his listener never before dreamed.

Surgery, problems of medicine, aye, even those of the cosmos itself, were robbed of their complexity by the magic touch of this master and brought within the comprehension of even the novice. No man more than he possessed the power, invaluable in a teacher, of seizing and fastening the attention of his hearers to whatever subject he presented. Some apparently simple and yet profound truth, presented to an audience as an aphorism, drew a ready acquiescence from all, and from that basis, step by step, the subject-matter was unfolded in an orderly sequence as forcible as it was logical.

That phase of the oratorical power which sways through the reasoning faculty rather than by the force of rhetoric amounted in him to almost a genius in itself. While none of the graces of language which stamp the polished littérateur were wanting in him, he scorned the mere play upon euphonism and sophistry which lend polish to and rob force from the discourses of many orators.

The induction of principles from accepted facts was his work, and how well it has been done is attested in the professional lives of thousands, and the gratitude of all who have come within the range of his influence directly or indirectly.

For a specific example of his mode of thought we may take his division of neoplasms into but two classes, those having an explainable and those an unexplainable origin. This generalization has given rise to the opinion among some of his critics that he was wanting in appreciation of the details of pathological anatomy, and yet one of the last conversations I had with that learned philosopher was upon this subject, and he discoursed upon the origins and metamorphoses of tumors with such a profound knowledge of details as would disillusion the most carping critic.

His simplicity arose from repletion, not from lack of knowledge. This very formulation has given hundreds of pupils an insight into a matter who, without it, might have been led into gross misconceptions, and no doubt in some cases into fatal error.

This is but one example; his thousands of former students are familiar with a multitude of others, which have served as sound and ever-present guides to them, avoiding through their application professional errors, and averting countless miseries to their patients.

One clinical teaching in particular is worthy of even more attention than it appears to have received, his method of operation for epithelioma. It was an axiom with him, that the greatest hope of cure in this malignant affection is in replacing the affected area by healthy tissue from another part, not the mere excision of the diseased structures. The records of his clinic show upon how sound a basis his opinion was formed. Not one fact amid the thousands he taught but was tabulated, classified to form part basis of some well-defined principle, and if a principle were already recognized was not placed in its proper relationship with its parent generalization.

His college chair reflected his mind; the other aspect of his teaching, his philosophical works, revealed the man himself. His philosophy was as near pure Platonism as any system could be and yet retain its distinctiveness. The Platonism of some two thousand years ago has speculation upon theoretic basis replaced by induction founded upon determined facts. Baconian lights are thrown upon Platonic pictures.

The Socratic method of exposition is applied throughout, and yet, contrary to the usual ending of a Platonic dialogue, definite answers to the problems discussed are presented.

There is not a school of philosophy from the Ionic to the Agnostic which has not been subjected to searching examination and criticism, and the results applied to an ethical code more than acceptable to Jew, Buddhist, Christian, or Mohammedan.

From the beaten track covered by all commentators he steps aside from the great mile-stones, Thales, Diogenes, Plato, Aristotle, Chrysippus, Epictetus, St. Augustine, Abelard, Averroës, Descartes, Bacon, Kant, Comte, and Spencer, to some almost forgotten thinker of the past, who has given one pearl, this Dr. Garretson has found with an unerring instinct and given it its proper position. To the contention of differing faculties, idealist and materialist, he gives his formulation,—"A thing is to the sense that uses it, what to that sense it seems to be."

For his personality, it was of such a type that those who knew him best loved him most. He represented the embodiment of all the reasons which caused the later Platonists to call Christianity Platonism.

Every trait of gentleness, affection, and a belief in the brotherhood of mankind, which distinguishes the true Christian, resided in him, and found expression in acts of his life known to but himself and the beneficiaries.

